# Property-Tuneable Microgels Fabricated by Using Flow-Focusing Microfluidic Geometry for Bioactive Agent Delivery

**DOI:** 10.3390/pharmaceutics13060787

**Published:** 2021-05-25

**Authors:** Wing-Fu Lai, Wing-Tak Wong

**Affiliations:** 1 Department of Applied Biology and Chemical Technology, Hong Kong Polytechnic University, Hong Kong, China; w.t.wong@polyu.edu.hk; 2 Ciechanover Institute of Precision and Regenerative Medicine, The Chinese University of Hong Kong (Shenzhen), Shenzhen 518172, China

**Keywords:** microgels, microfluidics, flow-focusing geometry, nutraceuticals, controlled release

## Abstract

Gelatine methacryloyl (GM) shows high biocompatibility and is extensively used in tissue engineering; however, few works have explored the use of GM in bioactive agent delivery. This study adopts a microfluidic approach involving the use of flow-focusing microfluidic geometry for microgel fabrication. This approach generates highly monodisperse microgels whose size can be tuned by altering various fabrication conditions (including the concentration of the gel-forming solution and the flow rates of different phases). By using tetracycline hydrochloride as a model agent, the fabricated microgels enable prolonged agent release, with the encapsulation efficiency being around 30–40% depending on the concentration of the gel-forming solution. Along with their negligible cytotoxicity, our microgels show the potential to serve as carriers of bioactive agents for food and pharmaceutical applications.

## 1. Introduction

Gelatine is a hydrolysis product of collagen [1,2,3], but compared to collagen, it shows less antigenicity and higher aqueous solubility [4]. While a gelatine solution can undergo gelation to form physical hydrogels upon a decrease in temperature, few derivatives of gelatine are capable of forming chemical hydrogels [5]. A good example is gelatine methacryloyl (GM), which is a derivative generated upon conjugation of methacrylate groups to amine-containing groups of gelatine. One favourable property of GM is its high tunability in properties, which can be manipulated simply by changing various synthetic and processing parameters [4]. In addition, the surface of GM supports cell adherence and growth [6]. This, along with the possibility of GM to undergo polymerisation in mild conditions, enables GM to be widely adopted as scaffolds for cell encapsulation [7,8,9]. In fact, the properties of GM as mentioned above are favourable not only for tissue engineering but also for bioactive agent delivery. Along with its polymerisability upon UV irradiation, GM can potentially be used to generate microgels via emulsion polymerisation as carriers of bioactive agents. 

Emulsion polymerisation is one of the commonly used approaches to generate microgels. During the process, high shear energy is applied to mix a gel-forming solution with an immiscible continuous phase to obtain emulsion particles for subsequent polymerisation reactions [10,11,12]. This method enables quick preparation of a large number of emulsion particles; however, the polydispersity of the generated microgels is high. Fragile bioactive agents (e.g., peptides and proteins) may also be damaged by high shear energy during the process of agent loading, which is generally performed concomitantly with the process of emulsion polymerisation. To address these problems, this study incorporates a microfluidic approach into the conventional process of emulsion polymerization to generate microgels for bioactive agent delivery. During microgel fabrication, an aqueous gel-forming solution flowing in one channel is subjected to the shear stress generated by the continuous oil phase flowing in another channel to produce microdroplets for subsequent photopolymerisation. By using flow-focusing microfluidic geometry, along with the manipulation of various fabrication conditions (including the concentration of the gel-forming solution and the flow rates of different phases), we have successfully produced microgels with different sizes and degrees of swelling for delivery of bioactive agents. 

## 2. Materials and Methods

### 2.1. Materials 

4-dimethylaminopyridine (DMAP), gelatine, and various other chemicals were purchased from Sigma-Aldrich (St. Louis, MO, USA). SU-8 2000 was purchased from MicroChem (Newton, MA, USA). Dulbecco’s Modified Eagle’s Medium (DMEM; Gibco, Grand Island, NE, USA), penicillin G-streptomycin sulphate (Life Technologies Corporation, Chicago, IL USA), and foetal bovine serum (FBS; Hangzhou Sijiqing Biological Engineering Materials Co., Ltd., Hangzhou, China) were used as the cell culture medium. Trypsin-EDTA (0.25% trypsin-EDTA) was obtained from Invitrogen (Carlsbad, CA, USA).

### 2.2. Synthesis of GM

7 g of gelatine and 0.35 g of DMAP were dissolved in 60 mL of dimethyl sulfoxide at 50 °C. Then, 3 mL of glycidyl methacrylate was added dropwise under constant stirring at 50 °C in an inert nitrogen atmosphere. After 48 h of reaction, the mixture was dialysed against deionized (DI) water for 3 days before lyophilisation to obtain GM.

### 2.3. Structural Characterisation

GM was solubilized in deuterium oxide (D_2_O). Proton nuclear magnetic resonance (^1^H-NMR) spectra were recorded using an NMR spectrometer (500 MHz; Bruker Corporation, Rheinstetten, Germany). The structures of gelatine, GM and MM were also characterized by using Fourier-transform infrared (FT-IR) spectroscopy (Nicolet5700; Thermo Nicolet Company, Waltham, MA, USA) at ambient conditions. Spectra were reported as an average of 16 scans.

### 2.4. Generation of Microgels by Using a Microfluidic Flow-Focusing Device

A silicon wafer was spin-coated with SU-8 2000 and baked at 95 °C. A photomask with patterns for microfluidic channels was placed on top of the wafer, followed by UV irradiation to crosslink the patterned area. The crosslinked photoresist was further solidified upon baking at 95 °C. After that, the wafer was put into the SU-8 developer, and was rinsed with isopropanol. A silicone elastomer base and a curing agent were mixed in a 10:1 ratio. The mixture was poured onto the master. Upon curing at 80 °C for 3 h, a polydimethylsiloxane (PDMS) elastomer with engraved microchannels was detached from the master. A hole punch (diameter = 0.5 mm) was adopted to produce the fluid inlet and outlet. A glass slide was irreversibly bonded to the PDMS elastomer upon surface treatment with oxygen plasma for 3 minutes to obtain a microfluidic device. 

During microgel fabrication, plastic tubing (inner diameter = 0.3 mm, outer diameter = 0.76 mm) was used to connect the inlet and outlet of the device. The aqueous phase was prepared by dissolving GM (5 or 8% (*w*/*v*)) and 2-hydroxy-4′-(2-hydroxyethoxy)-2-methylpropiophenone (0.2% (*w*/*v*)) in phosphate-buffered saline (PBS, pH 7.4)); whereas the oil phase was prepared by mixing Span 80 (20% (*v*/*v*)) with mineral oil (Sigma Aldrich, St. Louis, MO, USA). Different phases were injected into the device with controlled flow rates by using syringe pumps (PHD 2000; Harvard Apparatus, Holliston, MA, USA). Microgels were generated upon UV irradiation of the droplets generated in the device. They were retrieved by centrifugation, followed by washing with PBS five times. Microgels were designated as MM, with those fabricated from 5% (*w*/*v*), 6.5% (*w*/*v*) and 8% (*w*/*v*) GM solutions being designated as MM50, MM65 and MM80, respectively.

### 2.5. Determination of the Swelling Behaviour 

Microgels were immersed in PBS. Changes in the diameter of the microgels were recorded under an inverted optical microscope (Eclipse TE2000-U; Nikon, Tokyo, Japan) at regular time intervals and were analysed by using Image J software. 

### 2.6. Thermogravimetric Analysis (TGA)

TGA of gelatine, GM and MM was performed using a Q50 TGA analyser (TA Instruments, New Castle, DE, USA) equipped with platinum pans. Analysis was performed in an inert nitrogen atmosphere from 40 °C to 740 °C. The heating rate was set as 10 °C min^−1^.

### 2.7. Evaluation of Cytotoxicity

3T3 mouse fibroblasts and HEK293 cells were cultured as previously described [13]. The cells were seeded in a 96-well plate at a density of 5,000 cells per well. The plates were incubated at 37 °C under a humidified atmosphere of 5% CO_2_ for 24 h. Meanwhile, lyophilised MM80 was ground in DMEM using mortar and pestle to obtain a suspension with the desired concentration. The cell culture medium was replaced with 100 μL of the suspension. After 5 h incubation at 37 °C, the suspension in each well was replaced with the fresh cell culture medium. The CellTiter 96 Aqueous Non-radioactive Cell Proliferation Assay (MTS assay; Promega Corp., Madison, WI, USA) was performed according to the manufacturer’s instructions, either immediately or after 24 h of post-treatment incubation to determine the cell viability (%) in each well.

### 2.8. Determination of the Encapsulation Efficiency

Tetracycline hydrochloride was adopted as a model agent. To form agent-loaded microgels, MM50, MM65 and MM80 were prepared as usual but tetracycline hydrochloride was added to the aqueous phase at a concentration of 0.6% (*w*/*v*) prior to microgel fabrication. The concentration of unloaded tetracycline hydrochloride was determined using a ultraviolet-visible (UV-Vis) spectrophotometer (Cary 300; Varian, Palo Alto, CA, USA) at λ_max_ of 360 nm. The encapsulation efficiency (*EE*) was calculated using the following equation:

(1) $$EE\left(\%\right)=\frac{m_{l}}{m_{t}}\times 100\%$$

where *m_l_* is the mass of tetracycline hydrochloride encapsulated successfully by the microgels, and *m_t_* is the total mass of tetracycline hydrochloride added during the encapsulation process. 

### 2.9. Evaluation of the Kinetics of Agent Release

The release sustainability of the microgels was evaluated based on a previously reported protocol [14]. In brief, 1 g of lyophilised, agent-loaded microgels was placed in 10 mL of PBS (pH 7.4) and incubated at 37 °C. At regular time intervals, 1 mL of the release medium was withdrawn and replenished by the same amount of PBS. The amount of tetracycline hydrochloride released from the microgels was analysed using a UV-Vis spectrophotometer (Cary 300; Varian, Palo Alto, CA, USA) at λ_max_ of 360 nm. The percentage of cumulative agent release was calculated using the following equation:

(2) $$\mathrm{Cumulative}\;\mathrm{release}\;\left(\%\right)=\frac{\sum_{t=0}^{t}m_{t}}{m_{\infty }}\times 100\%$$

where *m_t_* is the mass of tetracycline hydrochloride released from the microgels at time *t*, and *m_∞_* is the total mass of tetracycline hydrochloride loaded into the microgels. The release curves were fitted into different kinetic models (including the zero-order model, the first-order model, the Higuchi model and the Korsmeyer-Peppas model) to analyse the mechanism of agent release.

### 2.10. Statistical Analysis

All data were presented as the means ± standard deviations of triplicate experiments. Student’s *t*-test was performed to assess the statistical significance. Differences with a *p*-value < 0.05 were considered to be statistically significant.

## 3. Results

### 3.1. Microgel Generation and Structural Characterisation

**Figure 1:**
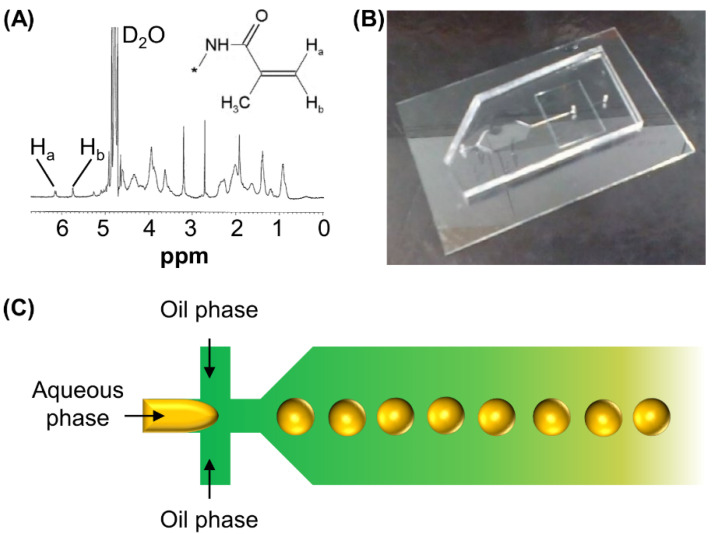
(**A**) ^1^H-NMR spectrum of GM. (**B**) A photo of the microfluidic device fabricated for microgel generation. (**C**) A schematic diagram depicting flow-focusing microfluidic geometry.

GM is a derivative of gelatine. The successful incorporation of methacryloyl substituent groups into gelatine was confirmed by ^1^H-NMR, in which the spectrum of GM shows peaks at 5.8 and 6.2 ppm (Figure 1A). These peaks were assigned to the acrylic protons of the grafted methacryloyl group. In addition, a peak was found at 1.9 ppm. This peak was contributed by the methyl proton signal of the grafted methacryloyl group. GM was adopted for the generation of microgels in a PDMS-based microfluidic device (Figure 1B). During microgel formation, a pre-gel solution containing GM and 2-hydroxy-4′-(2-hydroxyethoxy)-2-methylpropiophenone was used as the aqueous phase while mineral oil containing Span 80 was used as the oil phase (Figure 1C). 

**Figure 2:**
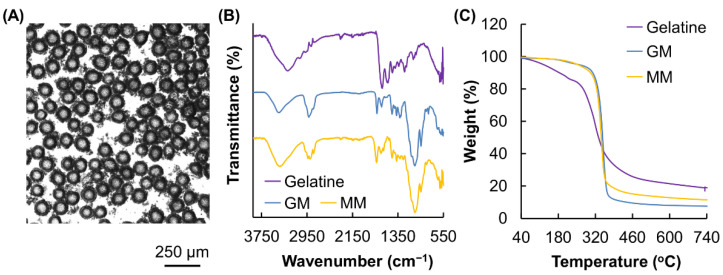
(**A**) A microscopic image of MM. (**B**) FTIR spectra of gelatine, GM and MM. (**C**) TGA curves of gelatine, GM and MM.

In the microfluidic device, the aqueous phase flowed through one channel and was intersected by the oil flow. A high concentration of Span 80 was adopted in this study to increase the viscosity of the oil phase so that adequate shear stress could be generated to produce aqueous droplets. In addition, the presence of a high concentration of Span 80 helped stabilise the aqueous droplets and prevent coalescence for the subsequent formation of monodisperse microgels for bioactive agent delivery (Figure 2A). The structure of the fabricated microgels, along with those of gelatine and GM, were characterised by using FT-IR (Figure 2B). In the spectrum of gelatine, a peak was found at around 1617 cm^−1^. This amide I band was attributed to C=O stretching vibrations of the amide group. In addition, a signal was detected at 1540 cm^−1^ and was assigned to N–H bending vibrations and C–H stretching vibrations. Similar peaks were also found in the spectrum of GM, which displayed characteristic bands at around 1631 cm^−1^ (amide I), 1556 cm^−1^ (amide II). In the spectrum of GM, a signal was detected at around 1720 cm^−1^. This signal was attributed to the carbonyl signal from the methacrylate group (C=O stretching) and was also found in the spectrum of MM. The methacrylate group in GM and MM was expected to be the functional group that enabled photo-crosslinking during microgel formation.

### 3.2. Thermal Properties and Size Control

The thermal properties of gelatine, as well as those of GM and MM, were characterised by using TGA (Figure 2C). The curve of gelatine exhibited two stages of weight loss. The first stage occurred below 220 °C, accounting for a weight loss of around 13.5%. This weight loss step was attributed to the loss of adsorbed moisture. The second stage occurred at the temperature range of 258–485 °C, leading to a weight loss of around 60%. In the curves of GM and MM, the percentage of weight loss in the first stage of the decomposition process from 50 to 220 °C was significantly reduced as compared to that of gelatine, suggesting an increase in the hydrophobicity of gelatine after structural modification. This is consistent with the observation made previously by Rajitha and colleagues [15], who modified gelatine with diethylenetriamine (DETA) and found that an increase in the hydrophobicity of gelatine leads to a decrease in the percentage of weight loss in the stage of moisture evaporation. 

**Figure 3:**
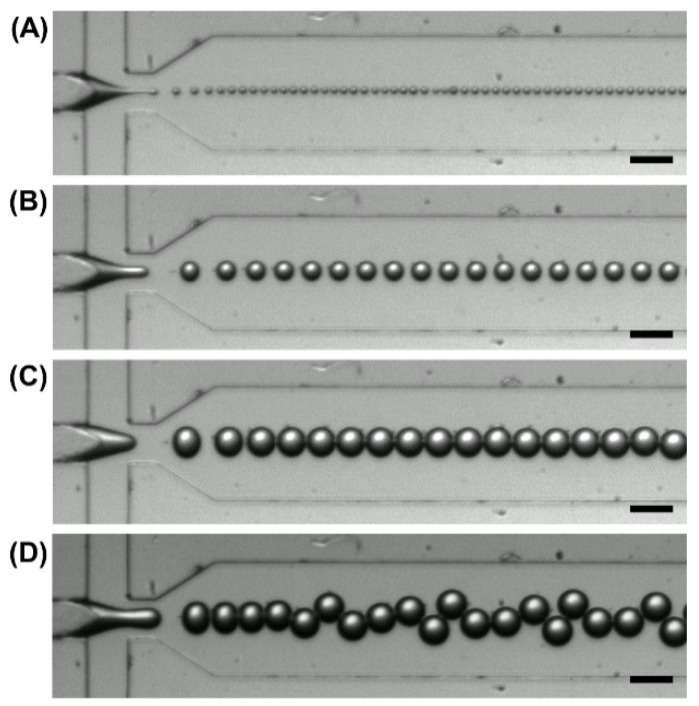
The size of MM fabricated at different *Q_aq_*/*Q_o_* values: (**A**) 0.05; (**B**) 0.1; (**C**) 0.2; (**D**) 0.3. The concentration of GM in the aqueous phase is 8% (*w*/*v*). Scale bar is 100 μm.

**Figure 4:**
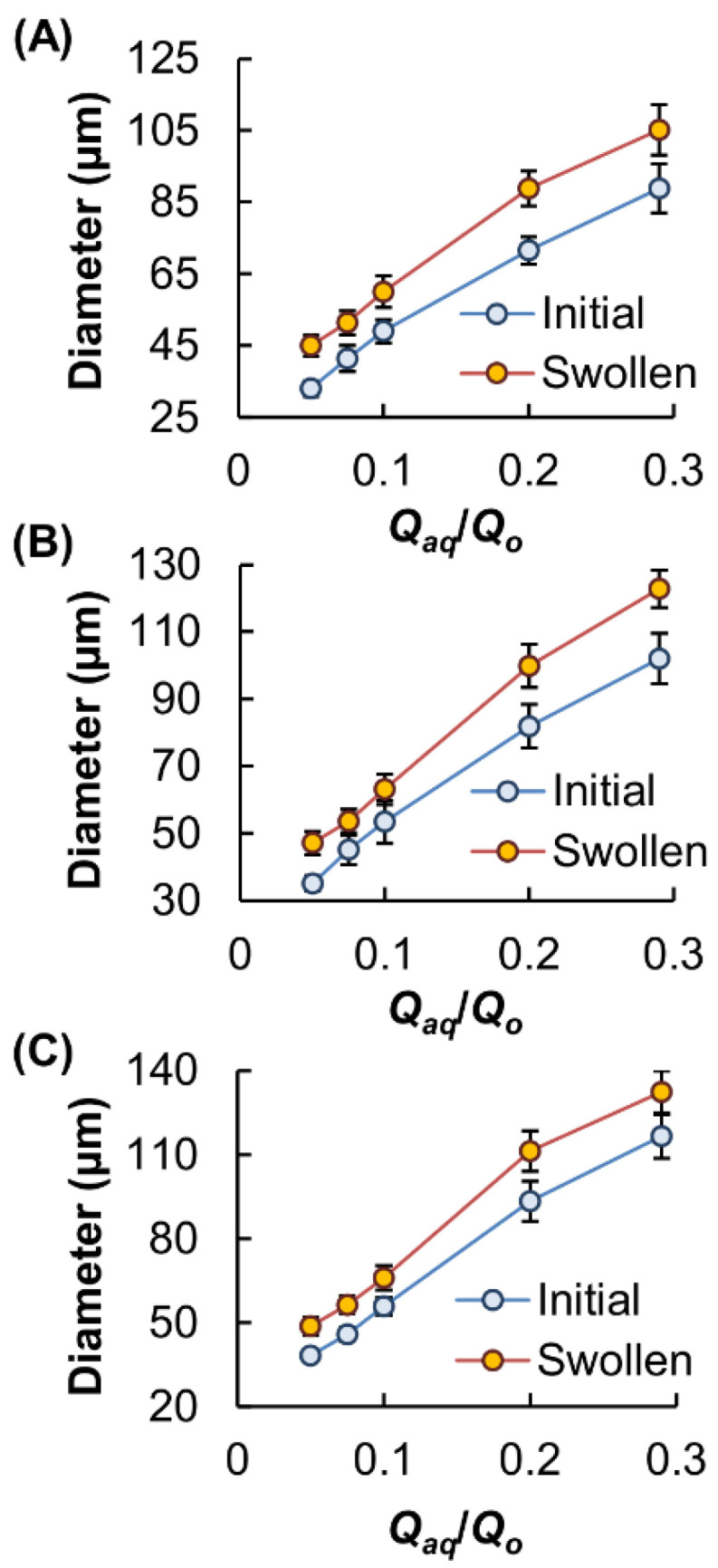
Changes in the average diameter of MM [(**A**) MM50, (**B**) MM65 and (**C**) MM80] upon changes in the *Q_aq_*/*Q_o_* value. The diameter was measured immediately after microgel fabrication and after swelling in PBS for 24 h.

**Figure 5:**
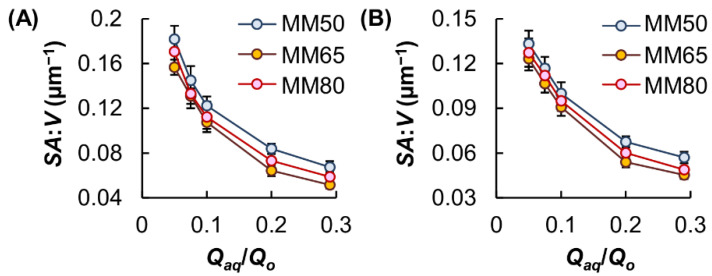
Changes in the *SA*:*V* value of MM upon changes in the *Q_aq_*/*Q_o_* value. The *SA*:*V* value was measured either (**A**) immediately after microgel fabrication or (**B**) after swelling in PBS for 24 h.

The use of a flow-focusing microfluidic device for microgel fabrication in this study enabled the generation of microgels with controlled size. The size of the microgels was manipulated by varying the ratio of the flow rates of the aqueous and oil phases (*Q_aq_*/*Q_o_*), and hence the magnitude of shear stress applied to the aqueous flow (Figure 3). Our results showed that the *Q_aq_*/*Q_o_* values are positively related to the microgel diameter (Figure 4) and are negatively related to the surface-area-to-volume ratio (*SA*:*V*) of the microgels formed (Figure 5). The concentration of GM in the aqueous phase also had a positive relationship with the size of the generated microgels. This was attributed to the fact that an increase in the GM concentration led to an increase in viscosity, leading to the formation of larger droplets. The size of the microgels further increased upon immersion in PBS, leading to a reduction in the *SA*:*V* values. 

### 3.3. Performance in Bioactive Agent Delivery

**Figure 6:**
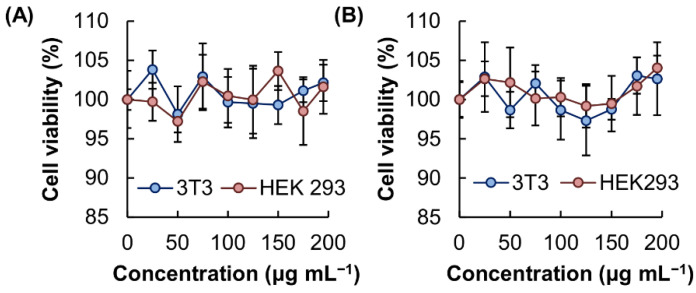
Viability of 3T3 fibroblasts and HEK 293 cells after 5 h treatment with MM, (**A**) before and (**B**) after 24 h post-treatment incubation.

In order to be used as carriers, the microgels have to possess a high safety profile [16,17,18,19]. The toxicity of the microgels was examined in vitro by using the MTS assay. No significant loss of cell viability was observed after 5 h treatment with the microgels. This indicated that the microgels have negligible acute cytotoxicity (Figure 6). To determine the chronic cytotoxicity of the microgels, the viability of the treated cells was further studied after 24 h post-treatment incubation. No detectable cytotoxicity was observed in all concentrations tested. This illustrated the negligible toxicity of the microgels for biological applications.

**Figure 7:**
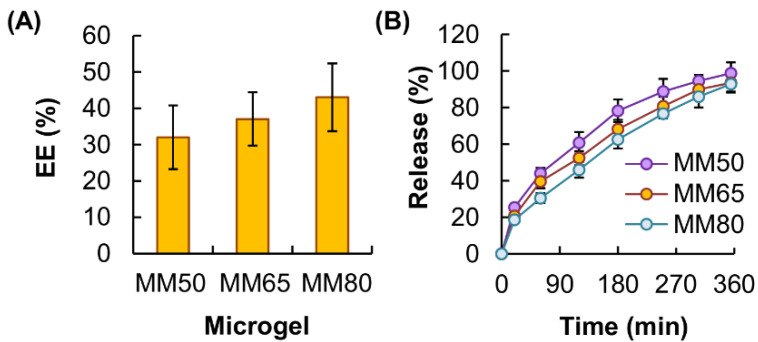
(**A**) The EE of MM50, MM65 and MM80. (**B**) Release profiles of tetracycline hydrochloride from MM50, MM65 and MM80.

To evaluate the performance of the microgels in bioactive agent delivery, tetracycline hydrochloride was adopted as a model agent. Depending on the concentration of GM in the microgels, the EE was estimated to be around 30–40% (Figure 7A). Among the microgels tested, MM80 displayed the highest sustainability of agent release (Figure 7B). This was partially attributed to the fact that, under the same *Q_aq_*/*Q_o_* value, microgels generated from a pre-gel solution containing a lower concentration of GM have a smaller size. Because a reduction in the particle size may cause a decrease in the diffusion length and an increase in the *SA*:*V* value [20,21,22,23], this favours the diffusion of the loaded molecules from the microgels to the surrounding medium and hence reduces the sustainability of agent release [24].

Upon fitting the curves of agent release into various kinetic models (including the zero-order model, the first-order model, the Higuchi model and the Korsmeyer-Peppas model) and based on the calculated regression coefficient (*r*^2^) values (Table 1), the release profiles of the agent-loaded microgels were found to fit the Higuchi model the most. This suggested that the process of agent release involves the penetration of the release medium into the hydrogel matrix. In addition, the release exponents (*n*), as calculated by using the Korsmeyer-Peppas’ equation, were 0.611, 0.516 and 0.461 for MM80, MM65 and MM50, respectively. This indicated that the release of tetracycline hydrochloride from the microgels is controlled by multiple processes (including polymer relaxation and agent diffusion), with anomalous non-Fickian transport being the major release mechanism.

## 4. Conclusions

Gelatine has a track record of applications in food, cosmetic and pharmaceutical industries [25,26,27]. To enhance the capacity of gelatine to form hydrogels, various derivatives have been generated over the years. One of them is GM, whose hydrogels have been widely adopted as scaffolds in tissue engineering [28,29,30]. In this study, we explored the potential use of GM in bioactive agent delivery, and adopted a microfluidic method to form monodisperse microgels. The size, as well as the *SA*:*V* value of the microgels, could be tuned not only by changing the *Q_aq_*/*Q_o_* value but also by manipulating the concentration of GM in the gel-forming solution. Such high tunability in properties, along with the high biocompatibility of GM, make our microgels worth being further exploited for food and pharmaceutical applications.

## Figures and Tables

**Table 1:** Correlation coefficients (*r^2^*) and release exponent (*n*) of different models for the kinetic data of agent release.

Model		Microgel		
		MM80	MM65	MM50
Zero-order model	*r* ^2^	0.912	0.832	0.758
First-order model	*r* ^2^	0.985	0.980	0.984
Higuchi model	*r* ^2^	0.986	0.998	0.994
Korsmeyer-Peppas model	*r* ^2^	0.998	0.998	0.996
	*n*	0.611	0.516	0.461

## Data Availability

Data are contained within the article.
